# Foraging Patch Selection in Winter: A Balance between Predation Risk and Thermoregulation Benefit

**DOI:** 10.1371/journal.pone.0068448

**Published:** 2013-07-12

**Authors:** Sara Villén-Pérez, Luis M. Carrascal, Javier Seoane

**Affiliations:** 1 Department of Biogeography and Global Change, Museo Nacional de Ciencias Naturales, Madrid, Spain; 2 Terrestrial Ecology Group, Department of Ecology, Universidad Autónoma de Madrid, Madrid, Spain; Hungarian Academy of Sciences, Hungary

## Abstract

In winter, foraging activity is intended to optimize food search while minimizing both thermoregulation costs and predation risk. Here we quantify the relative importance of thermoregulation and predation in foraging patch selection of woodland birds wintering in a Mediterranean montane forest. Specifically, we account for thermoregulation benefits related to temperature, and predation risk associated with both illumination of the feeding patch and distance to the nearest refuge provided by vegetation. We measured the amount of time that 38 marked individual birds belonging to five small passerine species spent foraging at artificial feeders. Feeders were located in forest patches that vary in distance to protective cover and exposure to sun radiation; temperature and illumination were registered locally by data loggers. Our results support the influence of both thermoregulation benefits and predation costs on feeding patch choice. The influence of distance to refuge (negative relationship) was nearly three times higher than that of temperature (positive relationship) in determining total foraging time spent at a patch. Light intensity had a negligible and no significant effect. This pattern was generalizable among species and individuals within species, and highlights the preponderance of latent predation risk over thermoregulation benefits on foraging decisions of birds wintering in temperate Mediterranean forests.

## Introduction

In the everyday struggle for life, wintering animals strive to meet the high energy requirements imposed by the season through a suite of behavioral strategies such as feeding during long periods of time and managing heat interchange with their environment [1]. Foraging patch selection can be viewed as a microclimate plus microhabitat selection process, being intended to optimize food search while minimizing both thermoregulation costs and predation risk. The risk of being predated may be related to multiple factors, among which the best known is the distance to a potential refuge against predators [2],[3],[4],[5],[6]. However, there are other factors determining predation risk; for instance, illumination levels highly influence the probabilities of being seen by a potential predator as well as the probabilities of detecting that predator in the surroundings [7],[8],[9].

Three-dimensional habitats such as forests create a complex sun-shade mosaic where each patch has a particular combination of sun incidence (and thus average temperature and light intensity), average food availability, and a given distance to the closer vegetation refuge from predators. Specifically, sun radiation is predicted to create a conflict in the decision-process of habitat use during foraging, due to the trade-off between energy savings by heating, and predation risk linked to high light intensity [7]. For instance, small passerines at low temperatures around zero °C reduce metabolic rate by approximately one half when exposed to sun radiation levels of ca. 1000 W/m^2^ and low wind speeds [10],[11]. Nevertheless, sun radiation may also increase predation risk through both higher probabilities of being detected by predators and reduced vision of prey due to glare effects [7],[8]. Glare effects may not affect prey and predators in the same way, as predators choose the direction of attack in order to reduce detection by prey [12]. On the other hand, habitat configuration influences the availability of refuges where prey may shelter against predators, and distance to protective cover determines the perception of predation risk. Cover functions as both refuge for prey and as concealment for predators, so birds devote more time to vigilance farther from refuge and prefer to feed near vegetation cover (e.g., [2],[6],[13],[14],[15]).

Habitat structure, thermal, food and predation factors are tightly related in the wild. For instance, temperature is related to food availability through arthropod activation and fruit productivity [16],[17], sun radiation determines both temperature and illumination levels, and it is at the same time influenced by the shade effect of vegetation cover. Due to their high level of correlation, the relative importance of these factors on foraging patch selection has rarely been assessed (but see [7],[18],[19]). Nevertheless, disentangling these intervening effects is crucial to understand the decision-making process of foraging patch selection in wintering animals.

Here we quantify the relative importance of thermoregulation benefits associated with ambient temperature, and predation risk linked to both illumination levels and distance to the nearest refuge, in foraging patch use by wintering forest birds. We analyze the influence of thermal benefits and predation risk in a montane Mediterranean forest of central Spain, with temperatures well below the thermoneutral zone [20],[21]. To attain this goal we made field observations under controlled and comparable situations according to foraging substrate, food access and food quality. We used specially designed feeders located on tree trunks at variable distance to vegetation cover and exposure to sun radiation, in such a manner that distance to cover was not associated with temperature and illumination levels. We registered temperature and light intensity with data loggers located at feeders, and used video recording to measure the cumulative time that individually marked birds exploited each feeder. This manipulative procedure under field conditions controls for the confounding effects of natural food availability, predation risk and thermoregulation benefit on foraging patch selection.

## Materials and Methods

### Study Area and Period

The study area was located in central Spain (‘El Ventorrillo’ field station, a 6 ha research facility of the Museo Nacional de Ciencias Naturales, CSIC; 1460 m a.s.l., 40°45′14″N 04°01′13″W, Sierra de Guadarrama, Madrid province), in a mixed forest dominated by Scots pine *Pinus sylvestris*, chestnut *Castanea sativa*, maples *Acer* spp., poplars *Populus nigra* and Pyrenean oak *Quercus pyrenaica*. Field work was carried out from 1 December 2010 until 10 February 2011. During the study, the average mean diurnal and night temperatures were 4.7°C and 1.8°C, respectively (average data from two data loggers –HOBO Pendant– placed in trunks at shade in the field station; see below), with a snowfall frequency of 22% days (data from the neighboring weather station of Navacerrada mountain pass, the nearest meteorological station, located at 1890 m a.s.l., 40°46’50″N 4°00’37″W). Day length at the winter solstice was 8.8 h.

### Study Species and Individual Identification

The study species were those belonging to the tree-gleaning guild of the mountainous forests of central Spain that have omnivorous diets [22]: *Cyanistes caeruleus* (Blue Tit, 10–12 g, own data), *Lophophanes cristatus* (Crested Tit, 10–12 g), *Sitta europaea* (Nuthatch, 23–25 g), *Parus major* (Great Tit, 18–20 g) and *Periparus ater* (Coal Tit, 9–10 g). These small-sized species mainly forage in the foliage, twigs and branches of trees, although they can also use the forest floor or trunks as foraging substrates (especially the Nuthatch, the Great Tit and the Crested Tit; [23]). In addition, they are regular exploiters of artificial feeders in the study region (e.g., [6]). Potential predators of small birds in the study area are the Sparrowhawk (*Accipiter nisus*) and the Tawny Owl (*Strix aluco*), although we did not see any of them during the marking or the sampling period. However, predation risk may be higher around feeders than elsewhere if predators are attracted there because of a higher abundance of prey. Birds typically react to predator presence by fleeing to the nearest vegetation cover (see [6],[24] for more details on previous study in the same field station).

In order to trap birds for individual identification, five rectangular funnel traps (40×40×60 cm) were placed all around the study area, 50–100 m apart and hanging from branches 1.5 m above the ground, from 1 December 2010 to 20 January 2011. This kind of trap is especially efficient at capturing tit species and can be used under adverse weather conditions [25]. Funnel traps were permanently baited with two cylindrical feeders, hanging from the trap roof, which contained husked peanuts. A translucent plastic sheet covered the roof of the traps to prevent birds from getting wet on rainy or snowy days. The escape doors of the traps remained opened on non-capture days, so birds could use the peanuts as a supplementary food source, but they were closed during the capture days from dawn to dusk. Trapping was conducted on 13 days from 8:30 to 17:30 hours GMT. On capture days, traps were inspected every 30–45 min. Captured birds were given unique color rings and released as quickly as possible after manipulation (handling time: 2–10 min). The Spanish agency in charge of environmental policy and animal welfare of Madrid (Consejería de Medio Ambiente, Comunidad de Madrid) approved the capture and study of birds (permit number 10/479348.9/10).

We marked a total of 60 individuals: 10 nuthatches, 17 great tits, 12 blue tits, 12 crested tits and 11 coal tits. Birds were recaptured an average of 6.45 times. Recaptures become steadily more frequent along the capture period, until reaching almost 100% of total captures in the five studied species.

### Experimental Set Up

Within the study area, ten feeders were established at least 20 m apart from one another. Each feeder was filled with husked peanuts and suspended from a nail 1.5 m above ground on thick trunks of pines or deciduous trees. Feeders were metallic cylinders (25×5 cm) made from a 4.8 mm mesh net allowing birds access to food. Feeders never ran out of peanuts during the study period (so birds could not find them empty). The feeders were located at least 10 m away from the positions where the funnel traps were located, so feeder choice by foraging birds could not be linked with previous experience related to predation risk due to bird trapping.

We carefully selected the location of the ten experimental feeders within the natural forest environment according to a broad variation in temperature, light intensity and distance to the nearest vegetation cover considering our previous experience in the study area (see Table 1).

**Table 1:** Characteristics of the ten experimental feeders.

Feeder	Temperature (°C)	Light intensity (lux)	Distance to cover (m)	Recording time (h:min)	Foraging time (s /10 h)	Visits to feeders (#/10 h)	# Species	# Individuals
**1**	4.8	781	0.1	44:05	772	18	5	22
**2**	4.5	299	0.3	42:11	1769	26	5	23
**3**	7.9	6645	0.3	46:11	3814	64	5	40
**4**	6.7	3685	0.3	53:26	916	17	5	24
**5**	5.9	2790	0.4	36:55	2997	56	5	35
**6**	5.7	2963	0.4	37:03	591	16	5	25
**7**	6.3	2453	3.2	41:07	1156	24	5	27
**8**	8.2	9910	3.4	26:17	306	6	3	11
**9**	5.6	2720	4.0	23:22	242	5	1	6
**10**	5.2	1000	6.5	26:24	116	5	3	4

Temperature and light intensity refer to those registered during the 14 sampling days. The dashed line divides feeders included in ‘near’ and ‘far’ categories. Feeding time and visits to feeders are cumulative numbers for all 38 birds, measured in seconds of feeder use and number of visits per 10 hours of recording time, respectively.

Air temperature and light intensity at each feeder was assessed with one data logger (HOBO Pendant, Onset; 58×33×23 mm) located at the lower end of each feeder. In order to standardize the registering of light intensity, the light sensor of the logger was pointed to the ground and a grey plastic plate of 60×40 mm was placed parallel to it and 2 cm below the light sensor. Thus, all loggers recorded the reflected light from same material of identical reflection properties. Data loggers recorded air temperature (measured in °C) and light intensity (measured in lux, lumens / m^2^) every minute during the sampling period. For each sampling day, 600 measurements of temperature and light intensity were obtained from 7:30 to 17:30 hours GMT. Data on temperature and light intensity during daytime at each feeder were averaged across the 14 days of sampling to characterize the environmental idiosyncrasy of feeders. Differences among feeding patches in average temperature ranged up to 3.7°C (from 4.5 to 8.2°C), while differences in average light intensity reached 9598 lux (from 299 to 9897 lux).

Two situations were selected to simulate low and high-predation risk locations according to the distance to protective vegetation cover, considered here as needles, small branches, twigs of bushes, and tree regrowth (<1 cm diameter) that could offer refuge to the birds when attacked by a predator. The low risk position (‘near’) was defined as at <0.4 m from the nearest vegetation cover, and the high-risk position (‘far’) as at >3 m from cover. Distance to cover is clearly associated with perceived predation risk, as previously found in similar studies reporting longer distances of escape to safe refuges, increases in vigilance rate, and decreases in average times spent at feeders per foraging bout in ‘far’ locations (e.g., [26] and references therein; [6],[13],[14],[15]). We selected six feeders ‘near’ and four ‘far’ from refuge, that cluster into two relatively homogeneous levels when considering the logarithm of their distances (ln distance to refuge in ‘near’ feeders: mean = 0.2, sd = 0.12; ‘far’ feeders: mean = 1.6, sd = 0.23;distance to refuge is considered on its logarithm according to the accelerated nature of flight movement while taking off). We made this unbalanced selection as the best *a priori* solution to uncouple the natural variation of distances and both temperature and light intensity at each feeder. Thus, the covariation between distance to vegetation refuge (‘near’ vs. ‘far’) and both average temperature and luminosity were minimal (temperature: F_1,8_ = 0.24, p = 0.635, R^2^ = 0.03; luminosity in logarithm: F_1,8_ = 0.34, p = 0.575, R^2^ = 0.04; n = 10 feeders using data in Table 1). A high correlation between temperature and luminosity was unavoidable when considering average figures for each feeder, as both variables are naturally dependent on sun radiation (R^2^ = 0.84). This correlation imposes a conflictive demand between the beneficial effects of temperature and the deleterious effects of luminosity.

### Feeder Use by Birds

Field work on feeder use was carried out from 21 January to 10 February 2011, considering only data for 14 cloudless, anticyclonic and near windless days (average wind speed of 3 m/s in the nearest weather station, Navacerrada mountain pass, located 4 km away from El Ventorrillo at 1890 m a.s.l. in an open, windier, mountain area; 40°46’50″N 4°00’37″W). Moreover, the influence of wind is considered to be negligible in our study design because feeders were located at low heights inside a dense, mature, forest (i.e., other trees provided shelter against wind, determining that wind direction was probably random in our study area). Average day length was 10 h 11 min and average potential sun radiation at zenith was 609 W/m^2^ during the sampling period. Artificial feeders were settled in the 10 study locations 32 days prior to the beginning of the sampling period, from 20 December 2010 to 20 January 2011. Considering this pre-feeding period, and the fact that the funnel traps also contained similar feeders with husked peanuts, we assumed the feeder setup was easily identifiable as a food source for birds.

We quantified the cumulative time foraging at each one of the 10 feeders by video monitoring (Table 2). A digital zoom video camera (Sanyo VPC-GH1 and Toshiba Gigashot GSC-K80) mounted on a tripod was placed at a distance of 1–2 m from each one of the 10 feeders, recording the number and duration of visits each individual bird made at each feeder. Frame rates were set at 21 fps. Color rings were clearly visible on video recordings, so it was possible to identify individual birds. The sampling period spanned from 7:30 to 17:30 hours GMT. Four video cameras were used in the sampling, rotating among the 10 of feeders, according to an *a priori* time schedule that tried to sample the feeders with a complete overlap of days and hours. Each feeder was videotaped for an average of 3.7 hours during a sampling day, and the average sampling effort was 37 h 42 min per feeder (range: 23 h 22 min / 53 h 26 min). Thus, each feeder was sampled approximately 23.1% of diurnal time available.

**Table 2:** Feeding time (seconds of feeder use per 10 hours of recording time) spent by individual birds at each feeder (see characteristics in Table 1).

Individual	Feeder #	Foraging time (s/10 h)
Cc-2	1	0
Cc-3	1	36.3
Cc-4	1	2.3
Cc-5	1	123.2
Cc-6	1	0
Lc-1	1	0
Lc-2	1	0
Lc-3	1	72.4
Lc-4	1	0
Lc-5	1	0
Pa-1	1	0.7
Pa-2	1	44.5
Pa-3	1	0
Pa-4	1	0
Pa-5	1	0
Pa-6	1	0
Pa-7	1	3.9
Pa-8	1	3.9
Pa-9	1	1.4
Pa-10	1	22.2
Pa-11	1	0.5
Pm-1	1	47.9
Pm-2	1	0
Pm-3	1	9.8
Pm-4	1	62.8
Pm-5	1	39.5
Pm-6	1	0
Se-1	1	90
Se-2	1	5.4
Se-3	1	0
Se-4	1	0
Se-5	1	0
Se-6	1	0
Se-7	1	159.5
Se-8	1	0
Se-9	1	17.7
Se-10	1	28.6
Cc-1	2	0
Cc-2	2	0
Cc-3	2	0
Cc-4	2	23.5
Cc-5	2	373.6
Cc-6	2	80.1
Lc-1	2	0
Lc-2	2	0
Lc-3	2	152.2
Lc-4	2	14.9
Lc-5	2	14.9
Pa-1	2	137
Pa-2	2	0
Pa-3	2	0
Pa-4	2	0
Pa-5	2	0
Pa-6	2	0
Pa-7	2	0
Pa-8	2	0
Pa-9	2	0
Pa-10	2	0
Pa-11	2	0
Pm-1	2	127.3
Pm-2	2	0
Pm-3	2	84.1
Pm-4	2	315.3
Pm-5	2	295.8
Pm-6	2	0
Se-1	2	32.9
Se-2	2	7.3
Se-3	2	39.1
Se-4	2	0
Se-5	2	8.8
Se-6	2	0
Se-7	2	26.1
Se-8	2	0
Se-9	2	27.3
Se-10	2	8.3
Cc-1	3	185.8
Cc-2	3	288.2
Cc-3	3	143.8
Cc-4	3	60.6
Cc-5	3	0
Cc-6	3	525.8
Lc-1	3	200.7
Lc-2	3	51.1
Lc-3	3	49.2
Lc-4	3	188.2
Lc-5	3	118.2
Pa-1	3	0
Pa-2	3	45.5
Pa-3	3	31.8
Pa-4	3	99.4
Pa-5	3	57.8
Pa-6	3	133
Pa-7	3	55.4
Pa-8	3	69.1
Pa-9	3	37
Pa-10	3	105.9
Pa-11	3	74.9
Pm-1	3	235.4
Pm-2	3	268.7
Pm-3	3	450
Pm-4	3	0
Pm-5	3	0
Pm-6	3	103.5
Se-1	3	13.4
Se-2	3	10.8
Se-3	3	24.7
Se-4	3	0
Se-5	3	33.8
Se-6	3	0
Se-7	3	0
Se-8	3	6.7
Se-9	3	68.6
Se-10	3	91.4
Cc-1	4	57.5
Cc-2	4	139.8
Cc-3	4	0
Cc-4	4	7.1
Cc-5	4	0
Cc-6	4	39.7
Lc-1	4	0
Lc-2	4	0
Lc-3	4	0
Lc-4	4	37.8
Lc-5	4	0
Pa-1	4	0
Pa-2	4	0
Pa-3	4	0
Pa-4	4	0
Pa-5	4	2.2
Pa-6	4	0
Pa-7	4	0
Pa-8	4	0
Pa-9	4	0
Pa-10	4	0
Pa-11	4	0
Pm-1	4	90.8
Pm-2	4	21.1
Pm-3	4	10.3
Pm-4	4	0
Pm-5	4	0
Pm-6	4	101.6
Se-1	4	1.5
Se-2	4	2.8
Se-3	4	9.2
Se-4	4	169.6
Se-5	4	16.3
Se-6	4	105
Se-7	4	3
Se-8	4	56
Se-9	4	25.8
Se-10	4	44
Cc-1	5	24.7
Cc-2	5	127.9
Cc-3	5	190.7
Cc-4	5	391.2
Cc-5	5	39.8
Cc-6	5	109.7
Lc-1	5	79.4
Lc-2	5	160.7
Lc-3	5	30.9
Lc-4	5	126.8
Lc-5	5	106.5
Pa-1	5	0
Pa-2	5	64.2
Pa-3	5	31.7
Pa-4	5	117.3
Pa-5	5	197.8
Pa-6	5	102.7
Pa-7	5	5.4
Pa-8	5	6.8
Pa-9	5	137.4
Pa-10	5	83.2
Pa-11	5	36.8
Pm-1	5	137.6
Pm-2	5	137.6
Pm-3	5	375.2
Pm-4	5	0
Pm-5	5	0
Pm-6	5	0
Se-1	5	34.4
Se-2	5	1.9
Se-3	5	0
Se-4	5	0
Se-5	5	0
Se-6	5	0
Se-7	5	92.9
Se-8	5	0
Se-9	5	46.1
Se-10	5	0
Cc-1	6	53.7
Cc-2	6	0
Cc-3	6	27.5
Cc-4	6	0
Cc-5	6	4.3
Cc-6	6	190.5
Lc-1	6	0
Lc-2	6	0
Lc-3	6	0
Lc-4	6	15.9
Lc-5	6	30.5
Pa-1	6	0
Pa-2	6	0
Pa-3	6	44
Pa-4	6	35.6
Pa-5	6	0
Pa-6	6	0
Pa-7	6	5.9
Pa-8	6	0
Pa-9	6	0
Pa-10	6	0
Pa-11	6	0
Pm-1	6	8.1
Pm-2	6	0
Pm-3	6	10.3
Pm-4	6	0
Pm-5	6	0
Pm-6	6	24.6
Se-1	6	4
Se-2	6	13
Se-3	6	25.1
Se-4	6	0
Se-5	6	51
Se-6	6	20.8
Se-7	6	0
Se-8	6	25.9
Se-9	6	0
Se-10	6	0
Cc-1	7	211.8
Cc-2	7	0
Cc-3	7	9.2
Cc-4	7	10
Cc-5	7	0
Cc-6	7	71.5
Lc-1	7	0
Lc-2	7	0
Lc-3	7	0
Lc-4	7	24.1
Lc-5	7	19.9
Pa-1	7	0
Pa-2	7	0
Pa-3	7	18.5
Pa-4	7	10.2
Pa-5	7	7.3
Pa-6	7	0
Pa-7	7	13.4
Pa-8	7	11.7
Pa-9	7	0.5
Pa-10	7	0
Pa-11	7	31.1
Pm-1	7	49.6
Pm-2	7	0
Pm-3	7	8.3
Pm-4	7	0
Pm-5	7	0
Pm-6	7	0
Se-1	7	48.6
Se-2	7	12.6
Se-3	7	100
Se-4	7	0
Se-5	7	268.7
Se-6	7	0
Se-7	7	32.1
Se-8	7	27
Se-9	7	55
Se-10	7	114.8
Cc-1	8	0
Cc-2	8	40.7
Cc-3	8	164
Cc-4	8	11.4
Cc-5	8	0
Cc-6	8	0
Lc-1	8	0
Lc-2	8	0
Lc-3	8	0
Lc-4	8	0
Lc-5	8	0
Pa-1	8	24.7
Pa-2	8	0
Pa-3	8	0
Pa-4	8	0
Pa-5	8	0
Pa-6	8	0
Pa-7	8	0
Pa-8	8	0
Pa-9	8	0
Pa-10	8	0
Pa-11	8	0
Pm-1	8	0
Pm-2	8	0
Pm-3	8	0
Pm-4	8	0
Pm-5	8	0
Pm-6	8	0
Se-1	8	8
Se-2	8	0
Se-3	8	26.3
Se-4	8	0
Se-5	8	0
Se-6	8	0
Se-7	8	10.7
Se-8	8	0
Se-9	8	14.5
Se-10	8	6.1
Cc-1	9	0
Cc-2	9	0
Cc-3	9	0
Cc-4	9	0
Cc-5	9	0
Cc-6	9	0
Lc-1	9	0
Lc-2	9	0
Lc-3	9	0
Lc-4	9	0
Lc-5	9	0
Pa-1	9	0
Pa-2	9	0
Pa-3	9	0
Pa-4	9	0
Pa-5	9	0
Pa-6	9	0
Pa-7	9	0
Pa-8	9	0
Pa-9	9	0
Pa-10	9	0
Pa-11	9	0
Pm-1	9	0
Pm-2	9	0
Pm-3	9	0
Pm-4	9	0
Pm-5	9	0
Pm-6	9	0
Se-1	9	12
Se-2	9	32.5
Se-3	9	0
Se-4	9	125
Se-5	9	0
Se-6	9	45.4
Se-7	9	21.8
Se-8	9	0
Se-9	9	5.1
Se-10	9	0
Cc-1	10	0
Cc-2	10	0
Cc-3	10	0
Cc-4	10	0
Cc-5	10	0
Cc-6	10	3.8
Lc-1	10	0
Lc-2	10	0
Lc-3	10	0
Lc-4	10	0
Lc-5	10	0
Pa-1	10	0
Pa-2	10	0
Pa-3	10	0
Pa-4	10	0
Pa-5	10	0
Pa-6	10	0
Pa-7	10	0
Pa-8	10	0
Pa-9	10	0
Pa-10	10	0
Pa-11	10	0
Pm-1	10	0
Pm-2	10	0
Pm-3	10	0
Pm-4	10	0
Pm-5	10	0
Pm-6	10	0
Se-1	10	0
Se-2	10	51.9
Se-3	10	0
Se-4	10	0
Se-5	10	0
Se-6	10	0
Se-7	10	0
Se-8	10	60.6
Se-9	10	0
Se-10	10	0

Only 38 individual birds for which at least 10 visits to feeders were obtained are shown (i.e., those included in the statistical analyses). Individuals are numbered within specie. Cc: *Cyanistes caeruleus*, Lc: *Lophophanes cristatus*, Se: *Sitta europaea*, Pm: *Parus major*, Pa: *Periparus ater.*

We used media player software (MicroSoft^®^ Windows Media Player 12 and VideoLAN VLC) to watch the video recordings and to measure the amount of time each individual bird spent foraging on each feeder.

The total number of visits to feeders was 900, with an average number of 23.7 visits that an individual bird made to the 10 feeders (range: 12–54), and an average time of stay per foraging bout of 58.9 seconds. The average number of different feeders used by each bird was 4.6, ranging between two and nine feeders. Some feeders were only visited on 11 occasions throughout the study period, while others accounted for more than 150 foraging bouts (a maximum of 276). The frequency of visits was significantly related to the cumulative time spent at each feeder throughout the sampling period (R^2^ = 0.83, p << 0.001). However, we chose the cumulative time spent at each feeder by each individual bird as the response variable, because it is a more precise measure of the foraging intensity at each feeder location as it includes the duration of all foraging bouts. Cumulative time spent at each feeder was standardized by dividing that amount of time by the recording time at each feeder, and it was expressed in seconds per 10 hours of recording.

We work with the cumulative time spent foraging at each feeder instead of the duration of each foraging bout because (1) we are interested in analyzing habitat use by a resident population of birds throughout the winter, (2) our study is not aimed at analyzing instantaneous decisions on how long to forage according with state-dependent conditions (e.g., internal reserves, satiation, time to dusk, etc.), and (3) the lack of feeding activity at some feeders by some birds is ecologically very relevant.

### Data Analyses

Although many ringed birds were detected in video recordings, only those individual birds for which at least 10 visits to feeders were obtained were included in the statistical analyses. We used this threshold considering that a minimum of 10 visits would be necessary for a bird to be able to forage at least once at each feeder. The final sample size was 38 different birds: 10 nuthatches, 6 great tits, 6 blue tits, 5 crested tits and 11 coal tits.

A General Linear Mixed Model was applied to analyze the cumulative time spent feeding at each feeder (response variable) by 38 different individual birds (i.e., a data matrix with cumulative times at ten feeders by 38 focal birds). Bird identity (BIRD) was considered as a random factor, species (SPECIES) and distance to cover (DISTANCE) as fixed factors, and average diurnal temperature (T) and average light intensity (LI) at feeders as covariates. Bird identity was nested within the corresponding species (i.e., differences among species were tested considering the individual bird as the sample unit instead of the foraging stays at each feeder). The mean square (MS) and the degrees of freedom (df) of the error terms were estimated following Satterthwaite’s method, which finds the linear combinations of sources of random variation that serve as appropriate error terms for testing the significance of the respective effect of interest. We also tested for parallelism in the relationships between time spent at feeders and temperature (DISTANCE*T) or light intensity (DISTANCE*LI) across the two levels of distance. The cumulative time spent foraging at feeders and light intensity were transformed logarithmically prior to data analyses. Homoscedasticity and normality of residuals of the General Linear Mixed Model were checked and they did not deviate from the canonical assumptions. Data were analyzed using StatSoft’s Statistica 10.0 (StatSoft Inc, Tulsa, Oklahoma).

## Results

**Figure 1:**
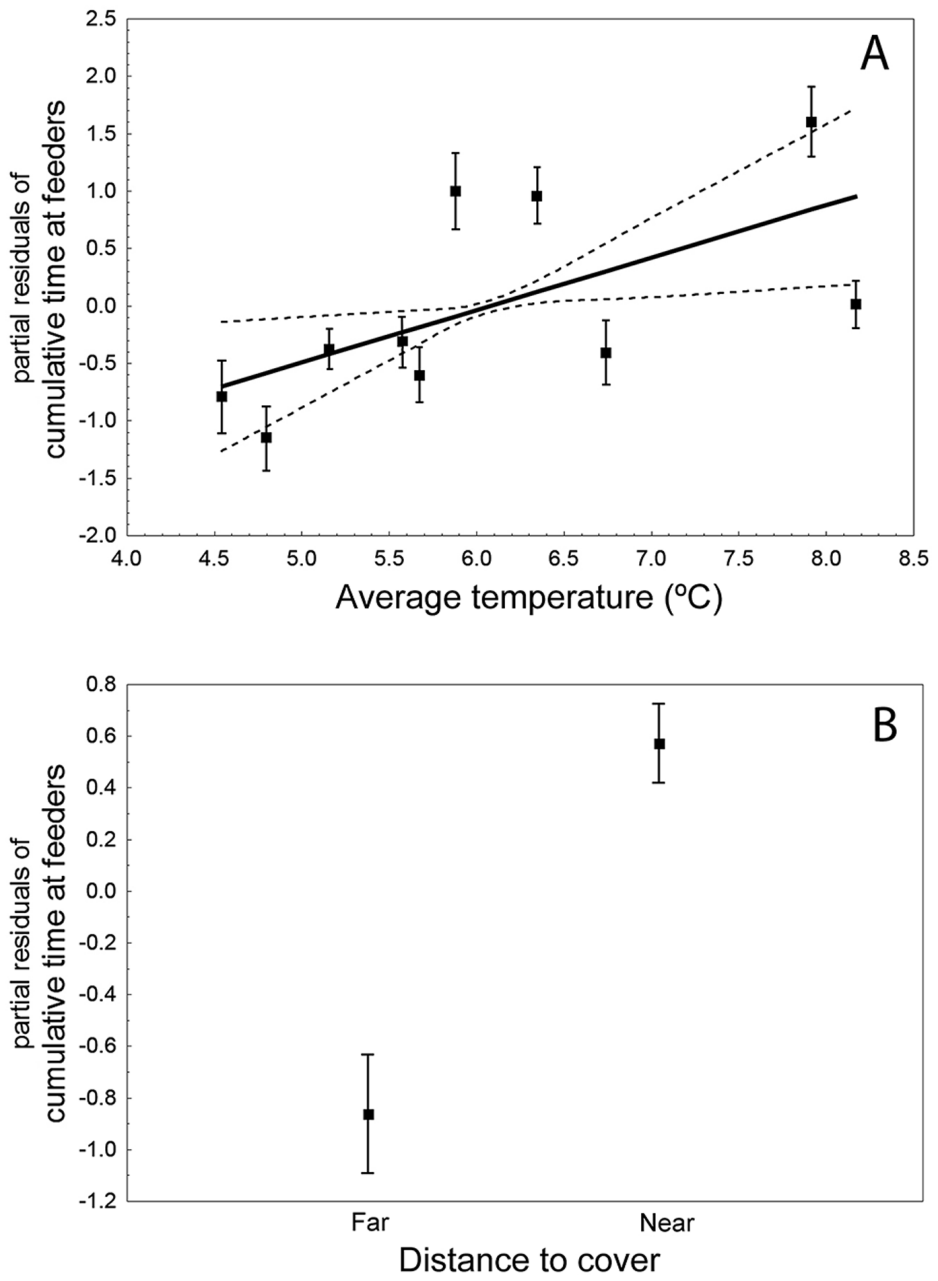
Influence of temperature and distance to cover on foraging patch use. Relationship between the cumulative time spent at each feeder by forest birds and (A) the average temperature at feeders along the study winter period (20 January–10 February 2011) and (B) the distance to the closest vegetation refuge against predators, in a mixed montane forest of central Spain. Figures represent average and standard error of partial residuals from a General Linear Mixed Model on ln seconds of stay / 10 sampling hours (Table 3) at all ten feeders (A) and at six far and four close feeders (B). Solid line represents linear fitting equation and dashed lines regression bands at 0.95 confidence level. Sample size is 38 individuals of five bird species: *Cyanistes caeruleus, Lophophanes cristatus, Periparus ater, Parus major* and *Sitta europaea.*

The General Linear Mixed Model (all effects: F_151,228_ = 1.41, p = 0.010, 48.2% of the variance accounted for; Table 3) shows that distance to cover (partial regression coefficient, β = −0.352) and temperature (β = 0.266) had significant effects on feeding intensity in the ten foraging patches (Figure 1). Birds spent more time feeding at ‘near’ (mean±se, 47.8±5.8 s/10 h, n = 38 birds) than at ‘far’ feeders (12.0±2.6 s/10 h), and at five warmer (48.6±6.6 s/10 h) than at five colder feeders (18.4±3.8 s/10 h). Neither the species nor the individual birds and the interaction terms birds×predictors reached significance (temperature, light intensity and distance to cover), which means that the described pattern of feeder use is generalizable among species and individuals within species. Moreover, the interaction terms between distance to cover and temperature (F_1,74_ = 0.302, p = 0.584) or distance×light intensity (F_1,74_ = 0.318, p = 0.574) were also non-significant, showing that the positive influence of temperature, or the lack of effect of illumination, did not change between near and far from vegetation cover. Distance to cover, which may provide refuge against predators, was the predictor variable with the highest magnitude effect (partial η^2^ = 0.66), followed by temperature at feeders (0.25). Therefore, the influence of distance to refuge was 2.6 times higher than that of temperature in determining the foraging intensity at feeding patches, while light intensity had a negligible and no significant effect.

**Table 3:** Sources of variation in foraging-patch use.

Effect	SS	Partial η^2^	df	F	p
Species	25.6	0.14	4, 33	1.40	0.257
Individual within species	151.5	0.16	33, 228	1.34	0.115
Temperature (°C)	24.4	0.25	1, 37	12.34	0.001
Ln light intensity (lux)	3.8	0.03	1, 37	1.33	0.256
Distance to cover (‘near’-‘far’)	179.3	0.66	1, 37	73.08	< 0.001
Individual×Temperature	73.2	0.09	37, 228	0.58	0.977
Individual×Ln light intensity	106.1	0.12	37, 228	0.83	0.741
Individual×Distance to cover	90.8	0.10	37, 228	0.71	0.890

General Linear Mixed Model of the cumulative time feeding at 10 foraging patches, by wintering birds (38 different individual birds belonging to 5 species) in a mixed montane forest of central Spain, considering bird identity (individual, random factor), species and distance to cover (fixed factors), and average diurnal temperature and average light intensity at feeders (covariates). Partial η^2^: partial eta-squared measuring the magnitude effects of predictor terms.

## Discussion

Wintering birds were able to identify small variations in temperature across foraging patches, devoting more time to relatively warm patches and likely reducing the thermoregulation expenditure while searching for food (Figure 1A). Birds also perceived the potential risk of being predated, spending more time foraging at safer patches with a close refuge available to escape from predators (Figure 1B). Noticeably, minimizing predation risk was much more important than reducing thermoregulation metabolic costs in these Mediterranean forests of relatively mild winter climate.

Wintering birds benefit from foraging at patches with higher environmental temperature where the costs of thermoregulation are reduced. Our results support this metabolic benefit in a forest scenario, where the sun-shade mosaic generates a broad spatial micro-variation in temperature [7],[27],[28],[29]. The energy saved by selecting the warmest foraging sites may be even more relevant when considering its cumulative effect on the long-term winter energy balance of these small birds, considering that they spend most of their winter daytime foraging under temperatures well below their thermoneutral zone ([20],[21]; 202 consecutive days below 20°C in winter 2010–2011 at Navacerrada mountain pass weather station; www.aemet.es).

In addition, birds that escape from predators by seeking shelter in dense vegetation minimize the risk of being predated by foraging close to vegetation cover [2],[4],[6],[30], and spend more time vigilant (even at large-habitat scale) in more fragmented or opened managed forest [31],[32]. Predation is thus perceived as a permanent potential risk and, as such, it exerts a continuous effect on the behavior of birds [33]. Moreover, predation is an unpredictable risk of ‘all or nothing’ consequences: a sole successful event of predation will be lethal, increasing this risk with distance to shelter. Therefore, small differences in flight time and distance to a safe refuge against predators while foraging have a paramount influence on microhabitat use. In our study, the average distances to the nearest refuge of ‘close’ and ‘far’ feeders were 0.3 and 4.3 m, corresponding approximately to 0.5 and 2.1 seconds of escape flights [34]. Our results show that birds follow anti-predator strategies steadily, even in the apparent absence of predators, as no predator attacks were observed during the ca. 420 hours devoted to fieldwork.

The effect of predation risk associated with the distance to dense cover was 2.6 times greater than that of the thermoregulation benefit associated with temperature (compare partial-η^2^ in Table 3). The hierarchical prioritization of predator avoidance over reduction of thermoregulation cost brings a sub-optimal exploitation of the thermal environment, as the time spent in patches with the lowest thermoregulation expenditure is not maximized [2],[7],[35],[36],[37]. An optimal exploitation of the thermal environment may gain importance in colder environments, and thus the relative importance of predation and thermoregulation is prone to change with the environment. In Mediterranean forests, in spite of the uncertainty associated with predation, betting on survival against predators preponderates over the tangible benefits of reducing metabolic costs.

We predicted that sun radiation would promote a conflictive demand between the benefits of sunbathing and predation risk associated with visibility. Contrary to our expectations, we found no effect of illumination determining the time that a forest patch is exploited. This contrast with some studies that relate luminance with both the risk of being detected by a predator and the difficulties the glare poses to detect predators [7],[8],[9],[38].

All species and all individuals within species followed a similar decision-making process in habitat use (see species, individual and interaction terms in Table 3). Therefore, the preponderance of avoiding predation over facilitating the maintenance of a positive energy balance is generalizable for small passerines facing the winter season, at least in relatively mild temperate Mediterranean forests. Thus, variations in the predation risk – thermoregulation trade-off would probably be related to the abiotic scenario rather than to the species involved. For this reason it would be interesting to test this trade-off in more restrictive scenarios according to winter climate.

In conclusion, wintering birds are able to identify and exploit subtle thermal variations in their foraging environment, minimizing the metabolic costs of thermoregulation while searching for food. Foraging intensity also depends on vegetation characteristics around feeding patches that define potential refuges against predators. The benefits of behavioral thermoregulation are direct, predictable and instantaneously perceived, but its quantitative importance is almost three times lower than that of reducing predation risk, which has an indirect and unpredictable effect.
